# Metal-organic framework-encapsulated dihydroartemisinin nanoparticles induces apoptotic cell death in ovarian cancer by blocking ROMO1-mediated ROS production

**DOI:** 10.1186/s12951-023-01959-3

**Published:** 2023-06-29

**Authors:** Yuanliang Yan, Xiaoxin Yang, Ning Han, Yuanhong Liu, Qiuju Liang, Liu-Gen Li, Jun Hu, Tong-Fei Li, Zhijie Xu

**Affiliations:** 1 Department of Pharmacy, Xiangya Hospital, Central South University, Changsha, 410008 Hunan China; 2 Hubei Key Laboratory of Embryonic Stem Cell Research, School of Basic Medical Sciences, Hubei University of Medicine, Shiyan, 442000 Hubei China; 3 School Institute of Chemical Biology and Nanomedicine, State Key Laboratory of Chemo/Biosensing and Chemometrics, College of Chemistry and Chemical Engineering, Hunan University, Changsha, 410082 Hunan China; 4 Department of Pathology, Xiangya Hospital, Central South University, Changsha, 410008 Hunan China; 5 National Clinical Research Center for Geriatric Disorders, Xiangya Hospital, Central South University, Changsha, 410008 Hunan China

**Keywords:** Dihydroartemisinin, Metal-organic framework, ROS production, Cell apoptosis, Ovarian cancer

## Abstract

Dihydroartemisinin (DHA), a natural product derived from the herbal medicine Artemisia annua, is recently used as a novel anti-cancer agent. However, some intrinsic disadvantages limit its potential for clinical management of cancer patients, such as poor water solubility and low bioavailability. Nowadays, the nanoscale drug delivery system emerges as a hopeful platform for improve the anti-cancer treatment. Accordingly, a metal-organic framework (MOF) based on zeolitic imidazolate framework-8 was designed and synthesized to carry DHA in the core (ZIF-DHA). Contrast with free DHA, these prepared ZIF-DHA nanoparticles (NPs) displayed preferable anti-tumor therapeutic activity in several ovarian cancer cells accompanied with suppressed production of cellular reactive oxygen species (ROS) and induced apoptotic cell death. 4D-FastDIA-based mass spectrometry technology indicated that down-regulated reactive oxygen species modulator 1 (ROMO1) might be regarded as potential therapeutic targets for ZIF-DHA NPs. Overexpression of ROMO1 in ovarian cancer cells significantly reversed the cellular ROS-generation induced by ZIF-DHA, as well as the pro-apoptosis effects. Taken together, our study elucidated and highlighted the potential of zeolitic imidazolate framework-8-based MOF to improve the activity of DHA to treat ovarian cancer. Our findings suggested that these prepared ZIF-DHA NPs could be an attractive therapeutic strategy for ovarian cancer.

**Supplementary Information:**

The online version contains supplementary material available at 10.1186/s12951-023-01959-3.

## Introduction

As one of most common types of gynecologic cancers, ovarian cancer is a major cause of death in female adults worldwide [1]. Several therapeutic strategies have been applied for clinic management of ovarian cancer, such as surgical intervention, radiochemotherapy and immunotherapy [2]. However, because of the diagnosis at advanced stages and treatment resistance, the patients with ovarian cancer frequently display the poor survival rates [3]. Thus, it is essential to explore novel therapeutic methods that could significantly improve the outcome of this disease.

Nowadays, the herb traditional medications have been confirm to serve as a viable alternative for cancer therapy [4, 5]. Artemisinin (Qinghaosu) is a kind of constituents isolated from herb *Artemisia annua*, exhibiting several proposed beneficial properties against human diseases. For a long time, artemisinin-based therapies have been widely used to treat the malaria in clinics [6]. More recently, emerging studies have pointed the underlying anti-cancer activities of artemisinin and its derivatives, although the detailed molecular mechanisms remain to be further elucidated. Jiao and colleague demonstrated that dihydroartemisinin (DHA), a derivative of artemisinin, displays a time- and dose-dependent cytotoxicity in ovarian cancer cell lines OVCA-420 and SKOV3 [7]. DHA administration significantly inhibited cell proliferative and improve chemotherapy sensitivity in multidrug-resistant lung cancer cells A549/DOX and A549/DDP [8]. However, the low solubility and poor bioavailability limited its therapeutic potential as a chemotherapeutic agent [9]. To overcome these problems, nanotechnology-based methods could be developed to enhance the clinical benefits of DHA in the treatment of cancers. Given that nanoparticulate systems could provide powerful strategies to carry anti-cancer drugs [10], it is desirable to design a simple and multi-functional nano-constructs for the efficient delivery of DHA to the tumors.

In the present studies, we developed a nanoscale metal-organic framework (MOF) based on zeolitic imidazolate framework-8 to carry DHA in the core (ZIF-DHA). Compared with free DHA, the resulting ZIF-DHA nanoparticles (NPs) evidently improved the cytotoxicity against ovarian cancer in vivo and in vitro. Following the mechanistic study, 4D-FastDIA-based mass spectrometry procedure was utilized to show that 11 co-upregulated and 7 co-downregulated proteins in ovarian cancer cells SKOV3 and A2780 after treatment with ZIF-DHA. Among these, reactive oxygen species modulator 1 (ROMO1) displayed the most significantly down-regulated expression. The following functional experiments indicated that ZIF-DHA suppressed cell growth and induced cell apoptosis through blocking ROMO1-mediated production of reactive oxygen species (ROS). These findings would provide valuable clues to impetus the clinical application of DHA in ovarian cancer therapy.

## Materials and methods

### Preparation of ZIF-DHA nanoparticles

The preparation of ZIF-DHA NPs was prepared by referring to previous related studies and improved [11]. Zinc nitrate hexahydrate 45 mg dissolved in 1.5 ml of deionized water. 2-methylimidazole 99 mg dissolved in 2.7 ml of methanol. DHA 5 mg dissolved in 0.25 ml of DMF. Aqueous zinc nitrate solution was added to the solution of 2-methylimidazole and DHA under room temperature with constant stirring and centrifuged at 12,000 rpm for 8 min to yield ZIF-DHA NPs, which were washed 3 times with methanol. The supernatant after the methanol wash was collected and treated with 0.2% aqueous NaOH at 50 °C for 30 min to convert the DHA into a UV absorbing compound for detection of its characteristic absorption peak by UV-Vis spectrophotometer, which can be calculated for unloaded DHA to assess the drug loading rate. Similarly, the ZIF and Methyl Red modified ZIF-DHA (ZIF-DHA-MR) were prepared with the same process. At last, these NPs were stored at 4 °C until further application.

### The characterization of ZIF-DHA nanoparticles

The morphology of ZIF-DHA NPs was viewed using transmission electron microscopy (TEM). Additionally, the hydrated size and zeta potential of ZIF-DHA NPs were assayed by a Malvern laser particle size analyzer. Crystal structure and functional groups of ZIF-DHA were detected using X-ray diffraction, and infrared spectroscopy to confirm the successful synthesis. ZIF-DHA was incubated with 5 mL of phosphate-citrate buffer (pH 5.2 or 2) respectively at 37 ℃ for 12 h. Their morphology was photographed with TEM in response to its degradation in an acidic environment. Moreover, the DHA and Zn^2+^ release from the ZIF-DHA NPs was also measured (PH = 7.4 and 5.2). The Fourier transform infrared (FTIR) spectrum was utilized to further reveal the new absorption peaks of DHA in the prepared ZIF-DHA.

### Cell Culture and cell transfection

Human ovarian epithelial cells IOSE80 and several ovarian cancer cells, SKOV3, A2780, OVCAR3 and TOV112D, were kindly obtained from Center for Molecular Imaging of Central South University, Xiangya Hospital. All these cells were cultured in Dulbecco’s Modified Eagle Medium (DMEM, HyClone Laboratories, United States) supplemented with 10% fetal bovine serum (FBS, Gibco, United States). The Flag-ROMO1 plasmids were purchased from Genechem. 293T cells were firstly seeded in 6-well plates, and co-transfected with the lentivirus package plasmids pMD2.G and psPAX2 for about 48 h. After then, the supernatants containing lentivirus were harvested. For cell transfection, the mixture of lentivirus and polybrene (5 µg/ml) was added into the culture medium of cancer cells.

### CCK-8 cell viability assay

The CCK-8 cell viability assay was performed according to the manufacturer’s introductions (b34304, Bimake, United States). In brief, about 1 × 10^3^ cancer cells were firstly plated in 96-well plates. After treatment with the indicated conditions, a spectrometer (PerkinElmer, United States) was used to detect the optical density of cells at 450 nm.

### Colony formation assay

To evaluate the functional roles of ZIF-DHA on cell growth, about 800 ovarian cancer cells were seeded in the 6-well plates. After 24 h, cells were treated with the indicated reagents, and incubated for about 15 days. 100% ethanol were used to fix the cell clones, and 0.006% crystal violet solution was used to stained the cell clones. After then, the colonies were counted using an automated counter.

### Analysis of ZIF-DHA’s cellular uptake and location

A2780 and SKOV3 cells in 24-well plates were incubated with ZIF-DHA (modified with PE) for 24 h. The fluorescence of ZIF-DHA was analyzed by flow cytometry (PE channel). Alternatively, the cells were seeded on a petri dish that was used for laser confocal microscopy before treating by ZIF-DHA. Then the cells were fixed with Paraformaldehyde before labeling with hoechst33342 and mitotraker (FITC-labeled). The co-location of ZIF-DHA and mitochondria in A2780 and SKOV3 cells were observed by confocal laser scanning microscopy.

### Molecular docking analysis of DHA’s binding effect with ROMO1

The prediction of ROMO1’crystal structure was identified in the AF-P60602-F1 database (https://www.uniprot.org/). ROMO1 was processed by PyMOL and then analyzed by Autodock for its power bonding with DHA. The most advantageous free power bonding was selected according to the principles described in auto-dock vina (http://vina.scripps.edu) & visualized using PyMOL.

### Cellular Thermal Shift Assay (CETSA)

To further verify the binding ability of DHA to ROMO1, a Cellular Thermal Shift Assay was conducted. Briefly, ovarian cancer cells were treated by DHA for 24 h before the protein extraction. The proteins were divided into 6 equal and heated at 46, 50, 54, 58, 62, and 66 ℃ for 4 min and cooled at 4 ℃ for 4 min, respectively. Then western blotting was applied to assay the expression of proteins. The thermal melting curves were plotted based on protein degradation.

### ROS assay

The DCFDA/H2DCFDA Kit (ab113851, Abcam, United States) was used to evaluate the cellular ROS concentration. After adding DCFDA Solution, the suspended cells were incubated away from light for about 30 min. Finally, a flow cytometer (BD Bioscience, CA, USA) was used to detect the fluorescence intensity in each sample.

### Cell apoptosis assay

The Annexin V-FITC Apoptosis Detection Kit (C1062S, beyotime, China) was used to evaluate the functional roles of ZIF-DHA on cell apoptosis. In brief, the ovarian cancer cells were washed and suspended in phosphate buffered saline (PBS). After adding Annexin V-FITC (5 µL) and propidium iodide (PI, 10 µL), the samples were incubated away from light for about 25 min. At last, a flow cytometer (BD Bioscience, CA, USA) was used to detect the apoptotic cells.

### Immunoblotting

For immunoblotting, ovarian cancer cells were lysed in RIPA lysis buffer with protease inhibitor cocktail. The total proteins in soluble fractions were usually collected by centrifugation at 12,000 rpm for 15–20 min. The equal quantity of total proteins was resolved on 10% SDS-PAGE and transferred to polyvinylidene fluoride (PVDF) membranes. After then, the PVDF membranes were incubated with the indicated antibodies, and visually analyzed by ChemiDoc XRS + imaging system (Bio-Rad, United States). The following antibodies were used in this study: anti-ROMO1 (1:1000, 24200-1-AP, Proteintech, United States), anti-ERK (1:1000, 11257-1-AP, Proteintech, United States), anti-phospho-ERK (1:500, 28733-1-AP, Proteintech, United States), anti-AKT (1:1000, 10176-2-AP, Proteintech, United States), anti-phospho-AKT (1:500, 66444-1-Ig, Proteintech, United States), anti-GAPDH (1:5000, 60004-1, Proteintech, United States) and anti-Actin (1:5000, sc-69,879, Santa Cruz, United States).

### In vivo xenograft models

As previously described [12], the xenograft models were established by the subcutaneous injection of 2 × 10^6^ A2780 ovarian cancer cells into the hind limb of female BALB/c (four weeks old) nude mice. When tumor volumes reached about 50 mm^3^, the mice were randomly divided into four groups (n = 5): (a) blank control groups, (b) ZIF groups, (c) DHA groups (10 mg/kg, once daily by tail vein injection), (d) ZIF-DHA groups. The tumor volume was calculated using the following equation: Tumor volume = length × width × width × 0.5. The tumor volume and body weight were measured every 3 days. The mice were closely monitored for 25 days and then euthanized. The tumors were collected, weighed, photographed, and used for the following experiments. All of the mouse experiments were approved by the Experimental Animal Welfare Ethics Committee of Central South University (No. CSU-2023-0149).

**Analysis of ZIF-DHA’s in vivo** **distribution**.

To investigate the blood circulation time and organ distribution, the whole blood samples were harvested from the mice treated with ZIF-DHA (1 dose, i.v.) at different points. Alternatively, the tumor grafts and vital organs from tumor-bearing mice treated with ZIF-DHA (q.d. 5 times, i.v.) were separated and obtained. The whole blood, tumor grafts, and vital organs were dissipated by aqua regia, wherein the concentrations of zinc ions were detected using Inductively Coupled Plasma Atomic Emission Spectrometry (ICP).

### Statistical analysis

All statistical data in this report were displayed as the mean ± standard deviation (SD). The difference between different groups were calculated with two-tailed Student’s t-test. These differences were statistically significant (*p < 0.05 and **p < 0.01).

## Results

### Characterization of the ZIF-DHA

**Fig. 1:**
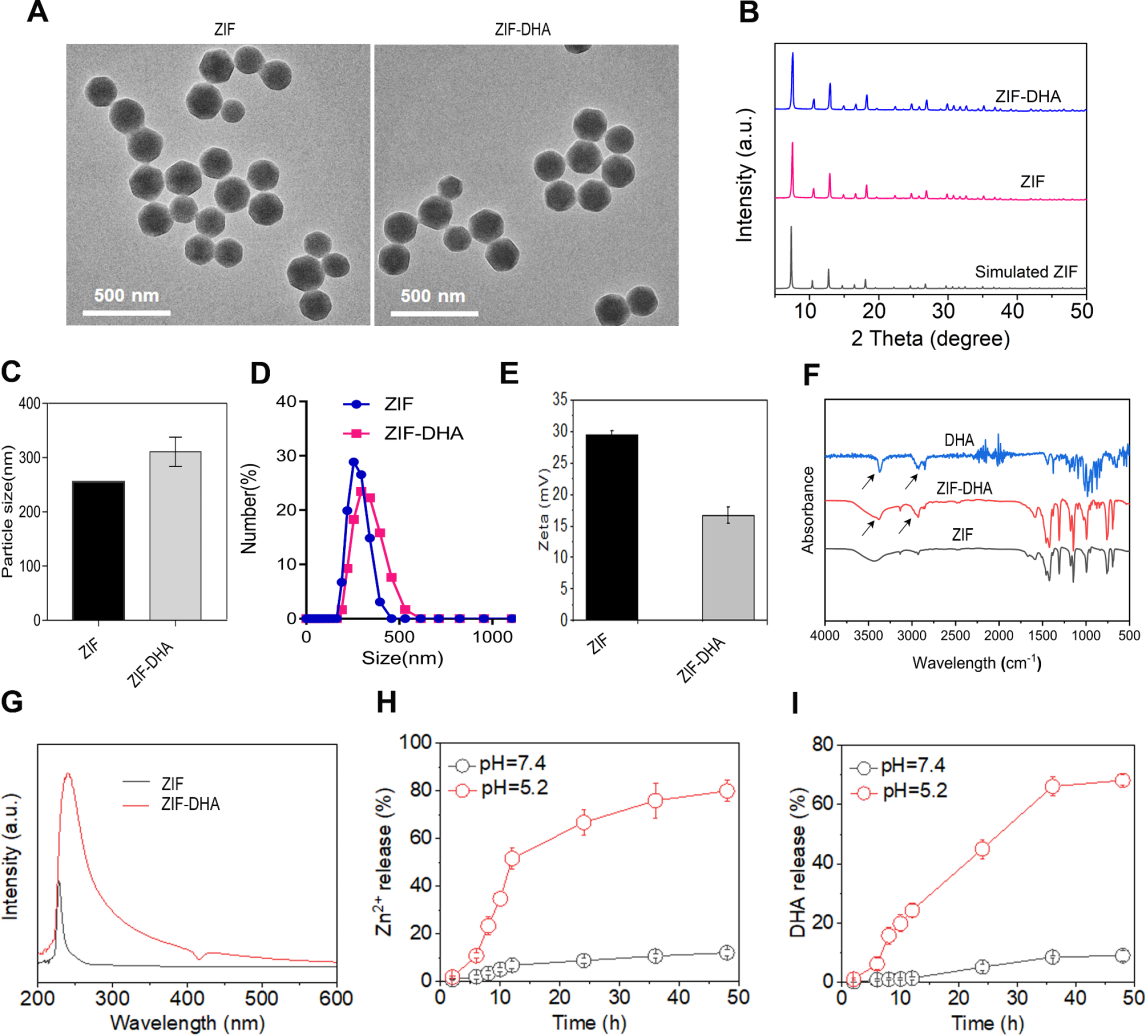
Characterizations of ZIF-DHA. (**A**) The TEM indicated the successfully prepared ZIF-DHA NPs. The particle size was about 100–150 nm. (**B**) PXRD experiments confirmed the ZIF pattern and crystal structure of ZIF-DHA. (**C-D**) The hydrodynamic size of ZIF-DHA was about 250 nm, as characterized by a Malvern laser particle size analyzer. (**E**) Zeta potential results of the prepared ZIF and ZIF-DHA. (**F**) The FTIR spectrum analysis of ZIF-DHA, ZIF, and DHA. (**G**) UV-vis detection suggested the effective encapsulation of DHA in ZIF-DHA. (**H-I**) The release of Zn^2+^ and DHA from ZIF-DHA at PH = 7.4 and 5.2

ZIF-DHA has a typical crystal structure with a size of 100–150 nm, as characterized by TEM in Fig. 1A. The crystallinity of ZIF-DHA was analyzed by the powder X-ray diffraction (PXRD), which matched well with the ZIF pattern (Fig. 1B). Further results showed that the average hydrodynamic size of ZIF and ZIF-DHA was about 250 nm along with good stability (Fig. 1C-D, Figure S1). Moreover, as presented in Fig. 1E, the zeta potential of the ZIF-DHA was also confirmed by Malvern zeta potential meter (positive charge), which was slightly decreased after DHA loading. FTIR detection of ZIF-DHA revealed some new absorption peaks in the range of 1000–1200, 2800, and 3400 cm^− 1^ compared with the spectrum of free ZIF NPs, which can be attributed to the stretching vibration peak of the O-O-C (peroxide) of DHA molecule in the pure form (Fig. 1F). The effective loading of DHA in ZIF NPs was further indicated by the featured 290 nm UV-vis peak (Fig. 1G). The loading efficiency of DHA on ZIF was about 19.25% according to the UV-vis detection, which was presented in Table S1. Moreover, the collapse of ZIF-DHA in the acid microenvironment was demonstrated in Figure S2, wherein the NPs lost the normal crystal structure constantly when PH = 5.2. The acidic environment (PH = 5.2) in cancer cells was also conducive for the release of Zn^2+^ and DHA from ZIF-DHA NPs (Fig. 1H-I). Taken together, these proofs spoke strongly that ZIF-DHA was successfully prepared and possessed the ability to respond to the acidic microenvironment in cancer cells.

### Evaluation of the anti-proliferative effects of ZIF-DHA on ovarian cancer cells

**Fig. 2:**
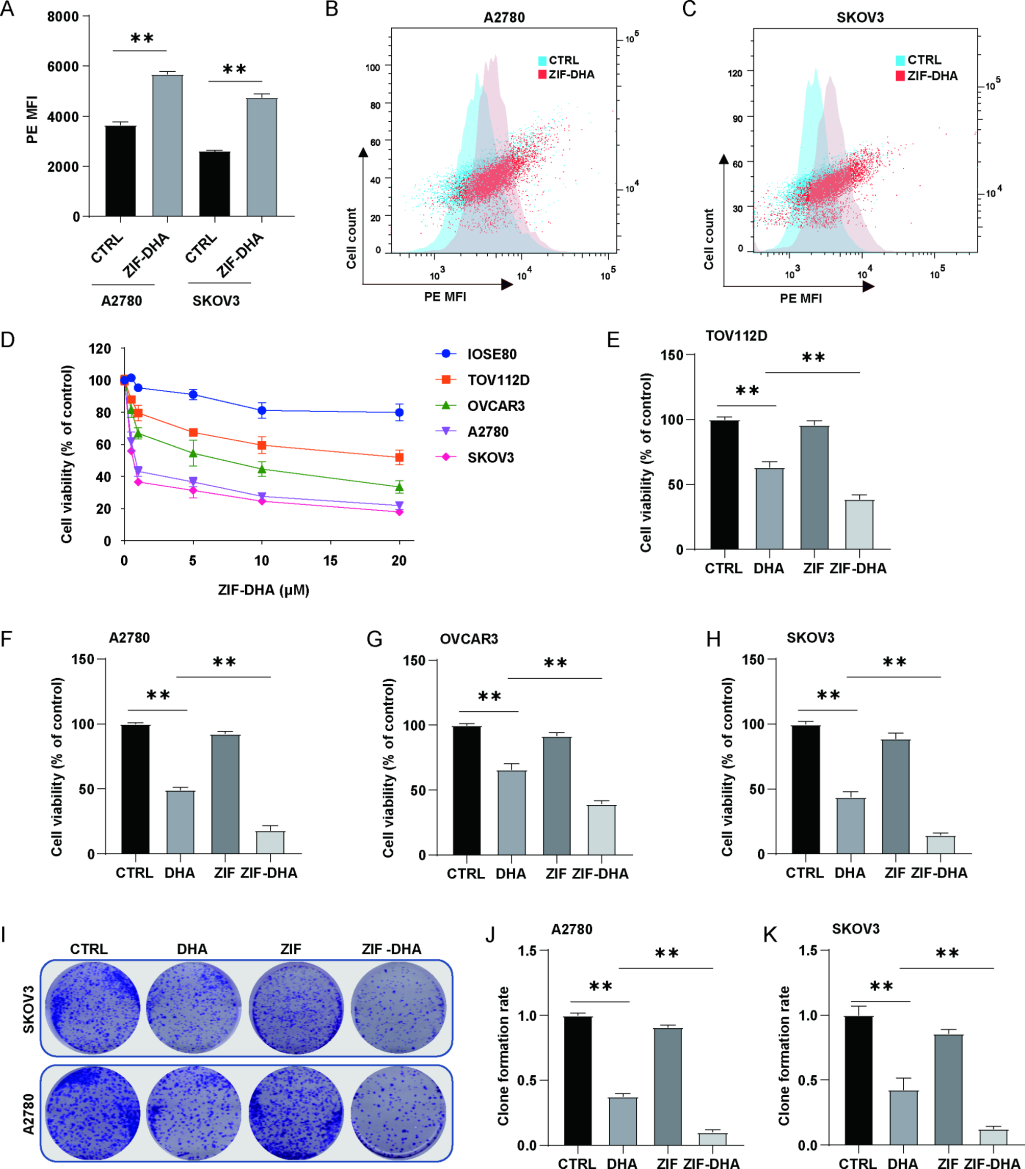
ZIF-DHA displayed the suppression of cell growth in ovarian cancer cells. (**A-C**) The intracellular uptake of ZIF-DHA-MR was detected using flow cytometry. (**D-H**) CCK-8 cell viability assay was used to explore the effects of ZIF-DHA on ovarian epithelial cells IOSE80 and several ovarian cancer cells, SKOV3, A2780, OVCAR3 and TOV112D. (**I-K**) The colony formation assay was used to evaluate the functional roles of ZIF-DHA on cell growth in ovarian cancer cells SKOV3 and A2780. The data in graphs represent mean ± SD. **p < 0.01

We performed the CCK-8 cell viability assay to explore the underlying cytotoxic effects of ZIF-DHA against human cancer cells. As shown in Figure S3, CCK-8 cell viability assay revealed the proliferation inhibition of ZIF-DHA against several cancer cells. Among these, the inhibition efficiency of ZIF-DHA was the most obvious in ovarian cancer cells. Moreover, the results of Fig. 2A-C showed that ZIF-DHA agents could be abundantly taken up by ovarian cancer cells, characterized by a significant increase in intracellular red fluorescence (ZIF-DHA-MR was labeled by red fluorescein as described in methods). Having established the effective obtain of ZIF-DHA by cancer cells, we will further investigate whether these NPs displayed significant cytotoxic effects against ovarian cancer cells. Expectedly, ZIF-DHA dose-dependently suppressed the cell growth rates in several ovarian cancer cell lines. However, administration of ZIF-DHA showed negligible toxicity against normal ovarian cell line IOSE80 even at relatively high concentrations (Fig. 2D). The cell survival rates of ovarian cancer cells in the ZIF-DHA-treated group were remarkably downregulated, even more than that of the free DHA-treated group, supporting the enhanced therapeutic efficacy (Fig. 2E-H). Similarly, higher anti-growth effects of ZIF-DHA compared with free DHA were further confirmed by the cell clonal formation experiment in ovarian cancer cells SKOV3 and A2780 (Fig. 2I-K). Collectively, these results indicated the good cytocompatibility and better anticancer efficiency of ZIF-DHA.

### Use of 4D-FastDIA to identify the altered pathways in response to ZIF-DHA

**Fig. 3:**
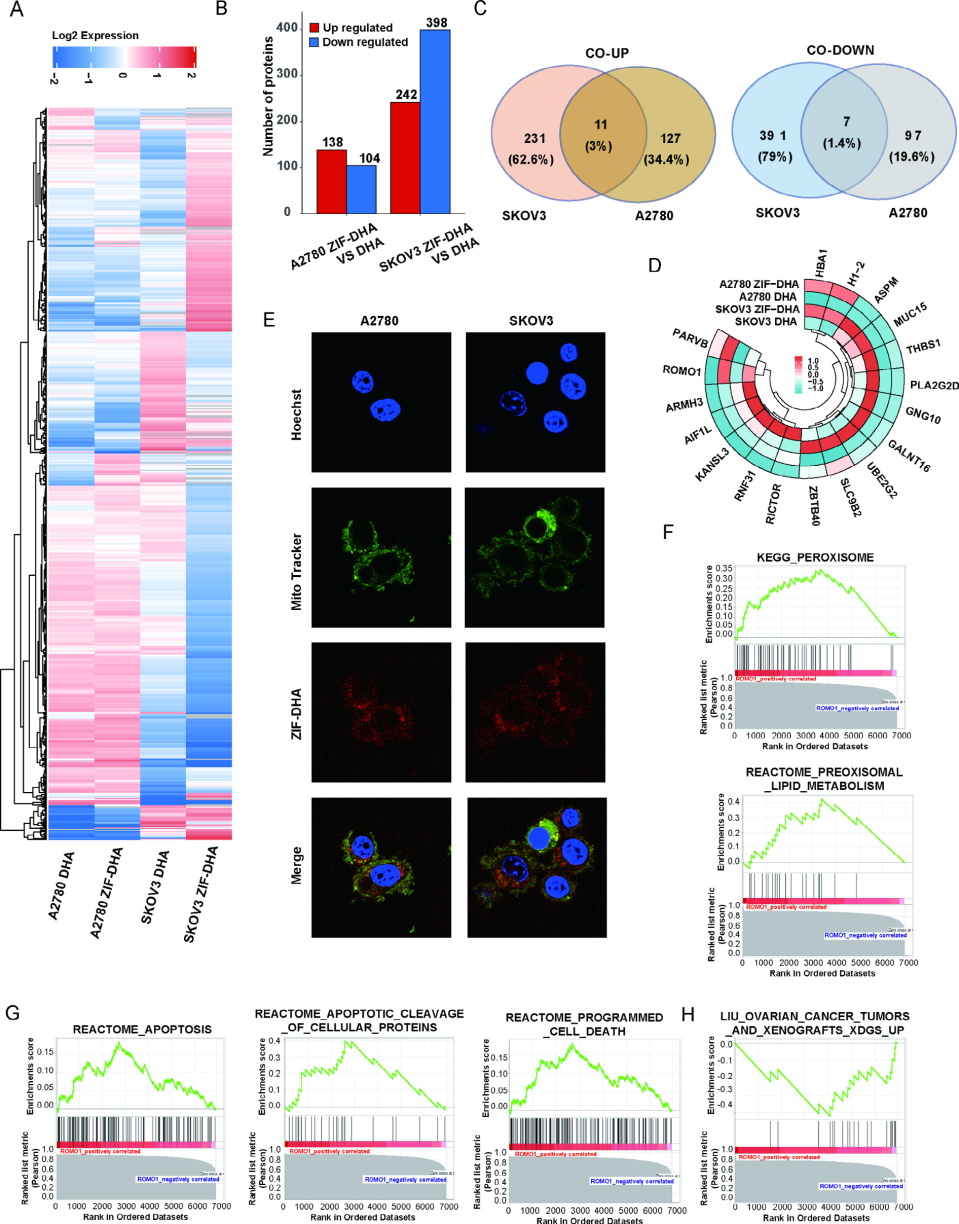
The altered pathways and molecules in response to ZIF-DHA identified by 4D-FastDIA. (**A**) The heatmap indicated the differentially expressed proteins in ZIF-DHA-treated SKOV3 and A2780 cells. (**B**) The graphs indicated the differentially expressed proteins in ZIF-DHA-treated SKOV3 and A2780 cells. (**C-D**) The co-differentially expressed proteins in ZIF-DHA-treated SKOV3 and A2780 cells. (**E**) mitochondria were labeled by the mito-tracker. The location of ZIF-DHA-MR were observed with laser confocal microscopy. (**F-H**) After ZIF-DHA treatment, GSEA indicated several biological pathways associated with ROMO1 expression

To explore the altered biochemical processes of ZIF-DHA treatment in the cellular proteome, ovarian cancer cells SKOV3 and A2780 were treated with ZIF-DHA and free DHA. After then, the total cellular proteins were trypsinized and identified by 4D-FastDIA-based mass spectrometry technology. Our analyses identified 56,854 peptides in approximately 6936 proteins that could be definitively quantified (Table S2). Using the screen criteria of |log(fold change)| ≥ 1.5, we quantified 242 differentially expressed proteins (138 up-regulated and 104 down-regulated) in ZIF-DHA-treated A2780 cells (Fig. 3A-B, Table S3). We also quantified 640 differentially expressed proteins (242 up-regulated and 398 down-regulated) in ZIF-DHA-treated SKOV3 cells (Fig. 3A-B, Table S4). Ultimately, utilizing the Venn diagrams, 18 proteins (11 up-regulated and 7 down-regulated) were observed to be co-differentially expressed in both ovarian cancer cells treated with ZIF-DHA (Fig. 3C, Table S5). The heatmaps revealed the most significantly down-regulated protein, ROMO1, might be regarded as potential therapeutic targets for ZIF-DHA (Fig. 3D). As reported, ROMO1 is primarily localized to the mitochondrial membrane and is a potent ROS regulator [13]. To explore whether the inhibition of ROMO1 by ZIF-DHA is associated with its large distribution with mitochondria, this was followed by an investigation of the co-localization of ZIF-DHA-MR with mitochondria. As displayed in Fig. 3E and Figure S4, the red fluorescence from the ZIF-DHA-MR and the green fluorescence from the mitochondria had an excellent overlap and co-localization efficiency, which meant that the agents were mostly allocated to the mitochondria. These data indicated that ZIF-DHA could be localized to the mitochondria in ovarian cancer cells.

Next, based on the mass spectrometry data, gene set enrichment analysis (GSEA) was performed to identify the underlying signaling pathways regulated by ROMO1 upon ZIF-DHA treatment. Accordingly, the c2.all.v2023.1.Hs.symbols in GSEA indicated several oxidation signaling pathways associated with ROMO1 expression, including peroxisome and peroxisomal lipid metabolism (Fig. 3F). The c2.cp.reactome.v2023.1.Hs.symbols in GSEA indicated the functional roles of ROMO1 in the apoptotic pathways, such as apoptosis, apoptotic cleavage of cellular proteins and programmed cell death (Fig. 3G). Moreover, the c2.all.v2023.1.Hs.symbols in GSEA further defined the important values of ROMO1 in ZIF-DHA-treated ovarian cancers (Fig. 3H). All these data collectively suggested the potential roles of ROMO1 in the regulation of oxidation signaling pathways and cell deaths after ZIF-DHA treatment.

### DHA had a direct binding effect with ROMO1

**Fig. 4:**
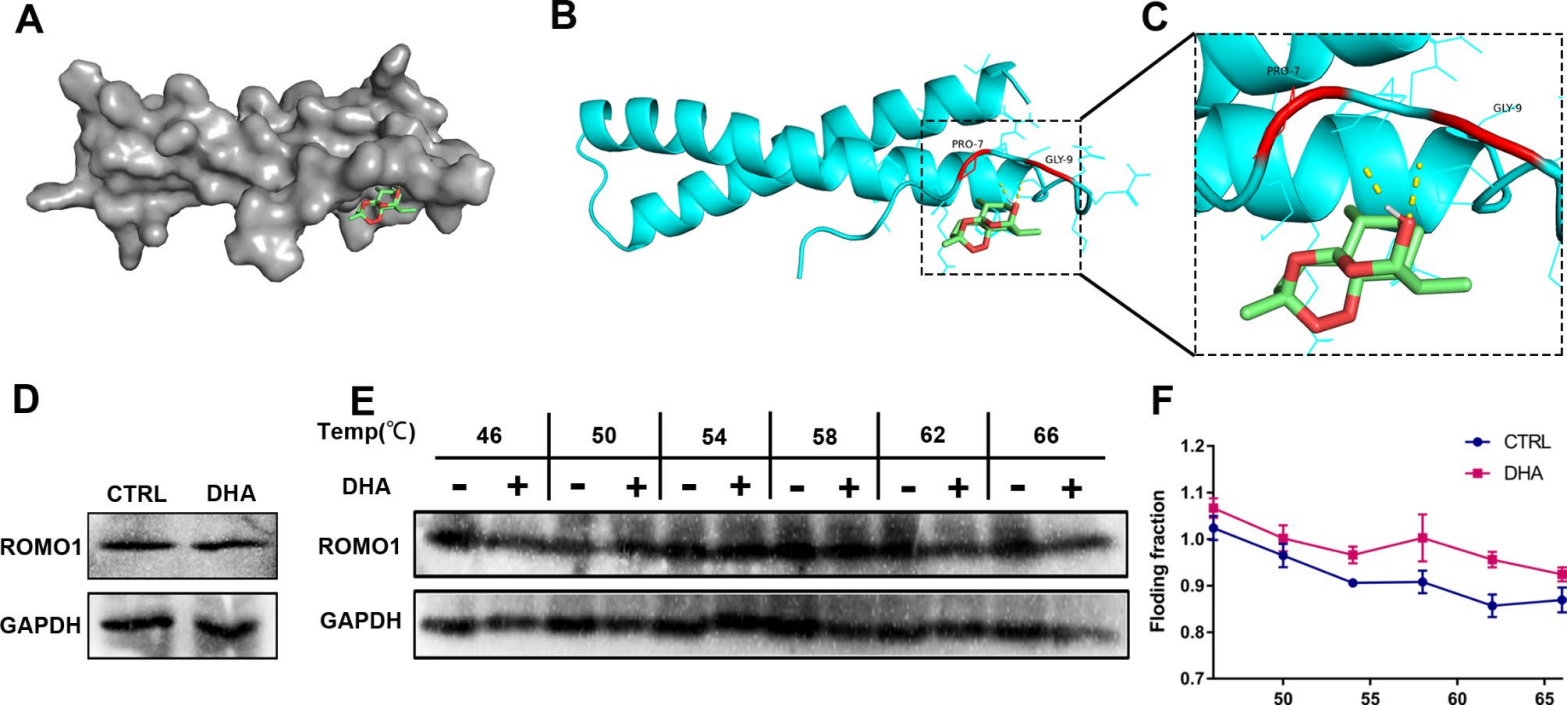
DHA could bind to ROMO1 but has no inhibitory effect per se. (**A-C**) The binding effect of DHA with ROMO1 was analyzed by molecular docking, wherein DHA may be linked with ROMO1 through PRO-7 and GLY-9. The power of the combination was − 5.1 kcal/mol. (**D**) Western blot indicated no obvious changes of ROMO1 expression after DHA treatment. (**E-F**) The CETSA experiment further turned out that DHA possessed a binding capacity to ROMO1.

In-depth analysis by molecular docking also revealed a possible interaction between DHA and the ROMO1 molecule in mitochondria. As presented, the predicted crystal structure of ROMO1 was acquired from the AF-P60602-F1 database (https://www.uniprot.org/). The structure of DHA was proceeded using PyMOL. The findings suggested that there was a possible mutual interaction force between DHA and ROMO1 (Fig. 4A-C). However, ROMO1 expression was only slightly suppressed in ovarian cancer cells with the DHA treatment (Fig. 4D). Next, the CETSA experiments were conducted thereupon to deeply analyze the effect of DHA on ROMO1. ROMO1 expression in ovarian cancer cells became more difficult to degrade, as evidenced by the reduced protein expression being mitigated and the shift of the thermal melting curve to the right (Fig. 4E-F). The above findings confirmed that DHA could bind to mitochondrial ROMO1 molecules, which itself had limited ability to inhibit these proteins.

### ZIF-DHA suppressed cellular ROS production and induced apoptosis through inhibiting ROMO1

**Fig. 5:**
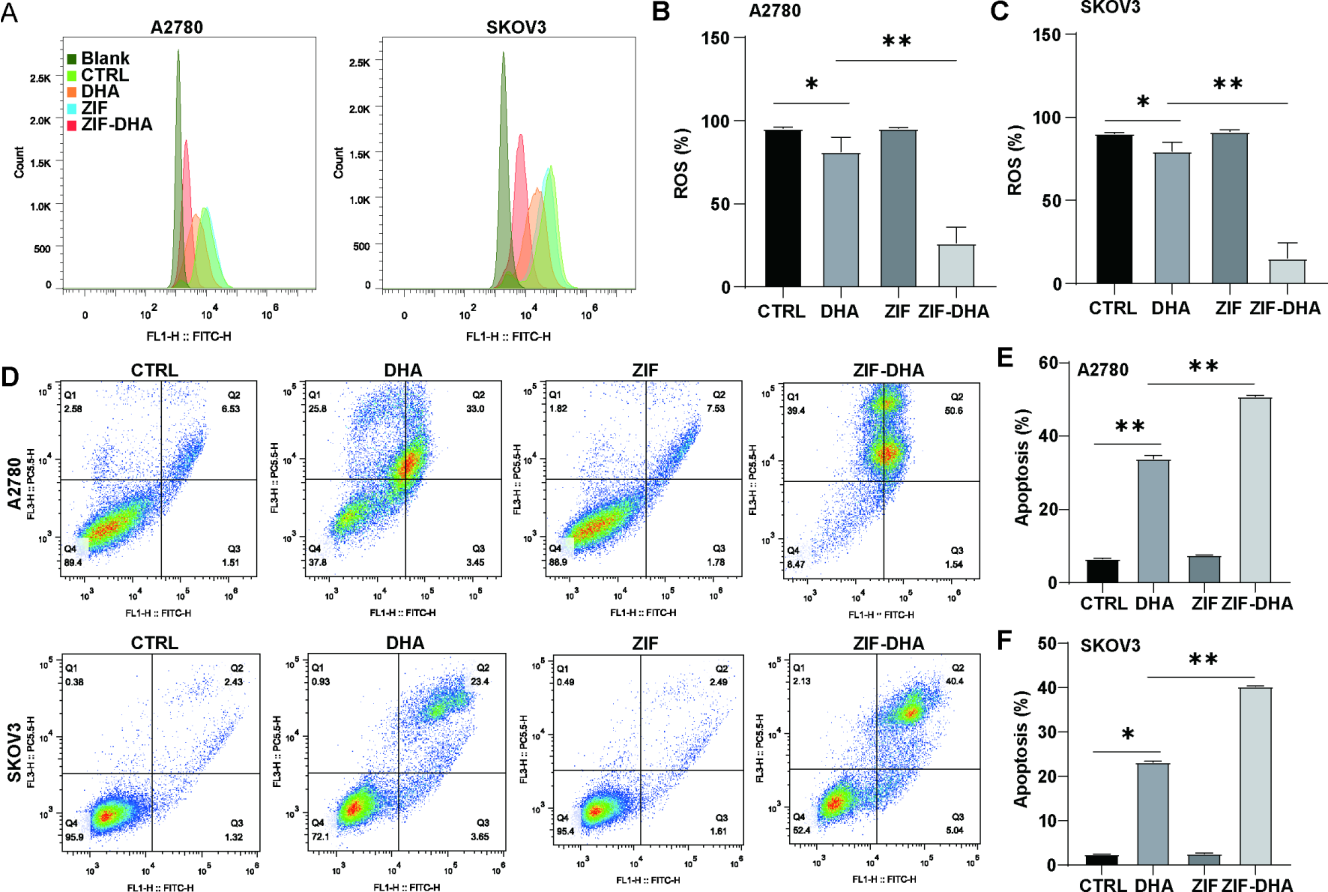
ZIF-DHA suppressed ROS production and generated cell apoptosis in ovarian cancer cells. (**A-C**) The DCFDA/H2DCFDA Kit was used to explore the effects of ZID-DHA on ROS generation in ovarian cancer cells SKOV3 and A2780. (**D-F**) The Annexin V-FITC Apoptosis Detection Kit was used to explore the pro-apoptosis effects of ZID-DHA in ovarian cancer cells SKOV3 and A2780. The data in graphs represent mean ± SD. *p < 0.05; **p < 0.01

Our afore-mentioned findings suggested the involvement of ZIF-DHA in controlling oxidation signaling, cell proliferation and apoptotic cell death. To test this hypothesis, we conducted the flow cytometry to analyze the changing trends of ROS generation and cell apoptosis in ZIF-treated cells. As shown in Fig. 5A-C, almost no changes of ROS levels could be found in the free ZIF-treated groups and control groups. Treatment with free DHA slightly suppress the cellular ROS concentrations in ovarian cancer cells SKOV3 and A2780. Interestingly, the strong inhibitory effects of ROS accumulation were observed upon ZIF-DHA treatment. Given that the aberrant ROS levels could lead to cell damage and eventual apoptosis in cancer [14, 15], we assessed whether ZIF-DHA displays the pro-apoptotic profiles. Expectedly, the ovarian cells treated with ZIF-DHA exhibited the strong pro-apoptosis effects, more than that of the free DHA-treated groups (Fig. 5D-F).

**Fig. 6:**
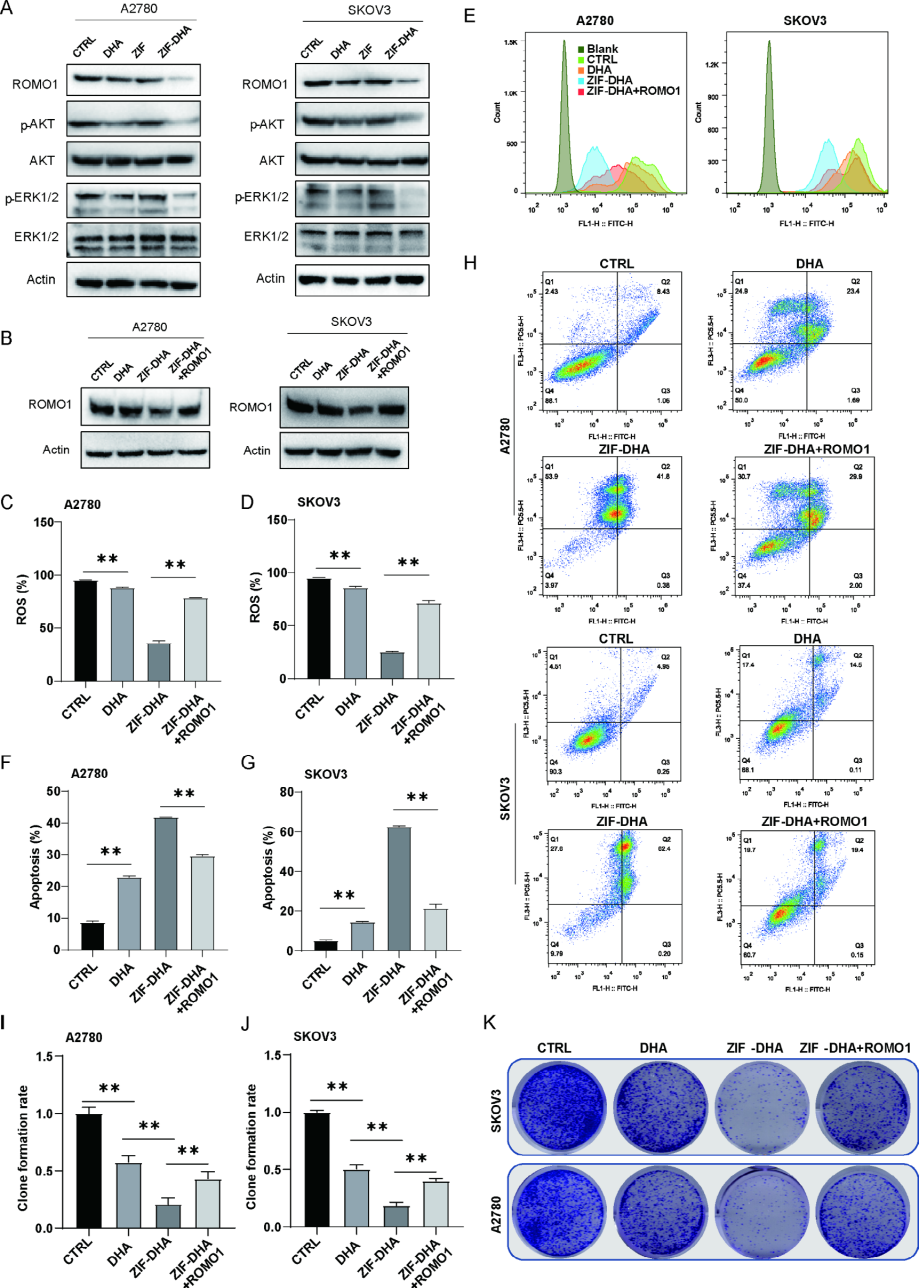
The cytotoxic effects of ZIF-DHA against ovarian cancer cells depended on ROMO1. (**A**) Treatment with ZIF-DHA reduced ROMO1 and its downstream signaling pathways. Ovarian cancer cells SKOV3 and A2780 were treated with free ZIF, free DHA or ZIF-DHA. Cell lysates were then blotted with indicated antibodies. (**B**) Ovarian cancer cells SKOV3 and A2780 cells stably expressing vector or Flag-ROMO1 were treated with ZIF-DHA. Cell lysates were then blotted with indicated antibodies. (**C-E**) The DCFDA/H2DCFDA Kit was used to explore the overexpression of ROMO1 in regulation of ZIF-DHA-induced ROS generation. (**F-H**) The Annexin V-FITC Apoptosis Detection Kit was used to explore the overexpression of ROMO1 in regulation of ZIF-DHA-induced apoptosis. (**I-K**) The colony formation assay was used to explore the overexpression of ROMO1 in regulation of ZIF-DHA-induced growth inhibition

Na and colleagues have found that depletion of ROMO1 could significantly inhibit the activation of extracellular signal-regulated kinase (ERK) and protein kinase B (AKT) signaling pathways, consequently suppressing the cell growth [16]. Consistently, we confirmed that ZIF-DHA treatment impaired ROMO1 expression and phosphorylation of ERK and AKT, two downstream factors of ROMO1, in SKOV3 and A2780 cells (Fig. 6A). After then, we applied overexpression of ROMO1 to determine its potential roles in regulation of ZIF-DHA-induced apoptosis. Administration of ZIF-DHA decreased the cellular ROS levels, which could be reversed by the ROMO1 overexpression (Fig. 6B-E). Subsequently, flow cytometry revealed that ectopic expression of ROMO1 in ZIF-DHA-treated SKOV3 and A2780 cells efficiently reversed the pro-apoptosis effects of ZIF-DHA (Fig. 6F-H). Upon ZIF-DHA treatment, we also observed an increased frequency of cell clonal formation in ROMO1 overexpressed SKOV3 and A2780 cells (Fig. 6I-K). All these results collectively suggested that ZIF-DHA NPs generated the apoptotic cell death with a significant decrease of ROMO1 in ovarian cancer cells.

### Tumoricidal activity and toxicity evaluation of ZIF-DHA in ovarian cancer models

**Fig. 7:**
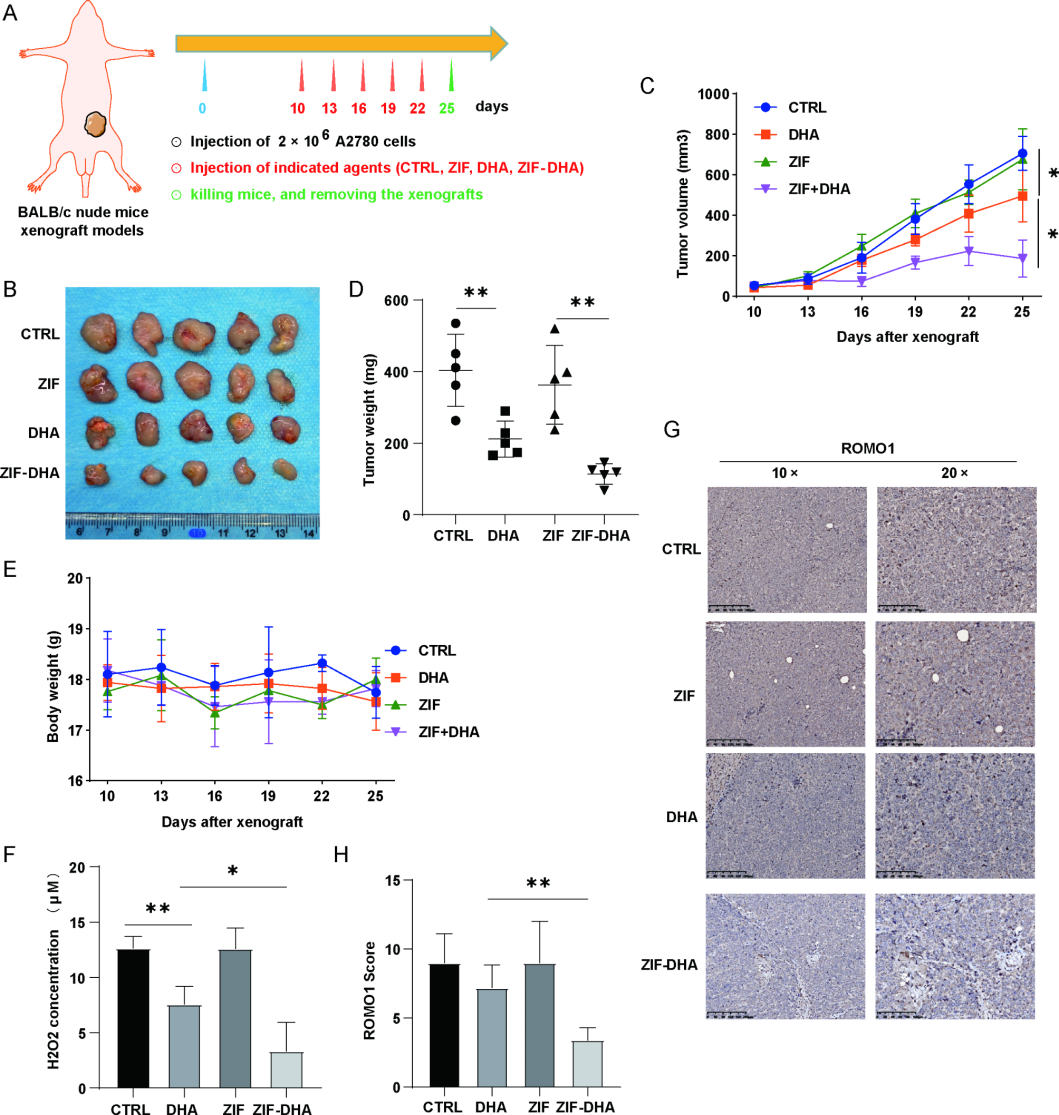
ZIF-DHA inhibited ROMO1 signaling in ovarian cancer xenografts in mice. (**A**) Schematic portraying the procedures of xenograft models. (**B**) Tumor tissues were harvested and photographed at the end of experiments. (**C**) The tumor growth curves in ovarian cancer xenografts treated with free ZIF, free DHA or ZIF-DHA. (**D**) The tumor weight in ovarian cancer xenografts treated with free ZIF, free DHA or ZIF-DHA. (**E**) The body weight curves in ovarian cancer xenografts treated with free ZIF, free DHA or ZIF-DHA. (**F**) ELISA assays were used to explore the effects of ZIF-DHA on cellular H_2_O_2_ concentration. (**G-H**) Immunohistochemical analysis was used to explore the effects of ZIF-DHA on the expression of ROMO1.

To better clarify the therapeutic potential of ZIF-DHA on ovarian cancer in vivo, the tumor-bearing mice were randomly divided into four groups: PBS control groups, free ZIF-treated groups, free DHA-treated groups and ZIF-DHA-treated groups (Fig. 7A). Firstly, the distribution of ZIF-DHA was evaluated, wherein the zinc ion content in tumor grafts and vital organs in the tumor-bearing mice was tested using ICP thereupon. As shown in Figure S5, the highest concentration of zinc ions was observed in the liver and spleen, followed by the tumor tissues, whereas the concentration of zinc ions was lower in other organs, indicating that ZIF-DHA could be enriched in tumors after intravenous injection. The preferred uptaken of ZIF-DHA by liver and spleen could reduce the potential off-target effects because the liver and spleen is rich in macrophages that effectively scavenge the nanoagents [17]. Furthermore, the results of ZIF-DHA’s blood circulation showed that the levels of zinc ions in the blood decreased slowly within 24 h after administration (Figure S6), evidencing that ZIF-DHA NPs might be slowly biodegraded in vivo. More importantly, as shown in Fig. 7B-D, compared with control groups, tail vein injection of DHA alone mildly impaired the tumor growth, whereas the ZIF-DHA injection could show the additive effects that inhibited the tumor growth. In addition, no obvious changes of body weight were found in the mice injected with ZIF-DHA (Fig. 7E), indicating the minimal side-effects of ZIF-DHA. The morphology of vital organs displayed no obvious changes (Figure S7), proving the little toxicity of ZIF-DHA in vivo. ELISA assays revealed that administration of ZIF-DHA remarkably decreased the cellular concentration of hydrogen peroxide (H_2_O_2_) (Fig. 7F). Immunohistochemical analysis confirmed that ZIF-DHA treatment significantly down-regulated the expression of ROMO1 (Fig. 7G-H). Thus, these findings demonstrated the safety and effectiveness of ZIF as a nanocarrier to improve the DHA anti-tumor effects.

## Discussion

**Fig. 8:**
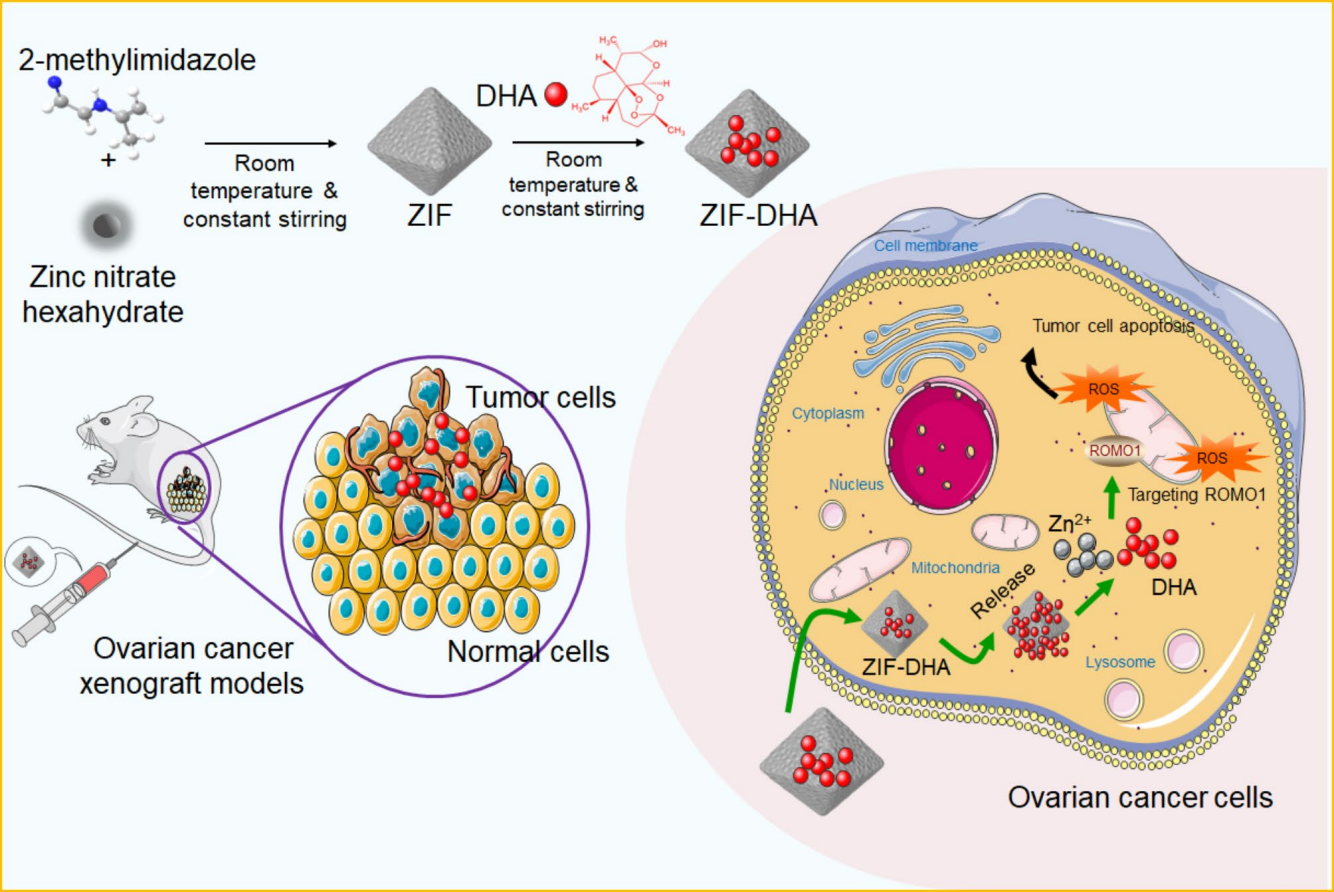
Schematic representation of ZIF-DHA nanoparticles against ovarian cancer. After tail vein injection of ZIF-DHA into the ovarian cancer xenograft models, the nanoscale ZIF-DHA could effectively down-regulated ROMO1, a mitochondrial membrane protein, consequently suppressing cell growth and inducing cell apoptosis through blocking ROMO1-mediated ROS production

Nowadays, because of specific anti-tumor properties and few side effects, the artemisinin derivative, DHA, is currently considered as the promising anti-cancer therapeutic drug [18]. Administration of DHA stimulated the DNA damage response and endoplasmic reticulum stress, simultaneously triggering ferroptosis and immunogenic cell death in lung cancer cells [19]. DHA could be used to improve the anti-cancer immunotherapy through remodeling tumor-associated macrophages into the M1-type phenotype [20]. However, several inherent disadvantages of DHA limit its therapeutic efficacy, such as poor water solubility, low stability, and short plasma half-life [21]. Thus, there is an urgent need to rationally design the DHA carriers to enhance the anti-cancer efficiency. In particular, several nanoscale drug carriers are already utilized to maximize the therapeutic efficacy of DHA. Huang et al. [22] developed Fe^3+^-doped manganese dioxide nanosheets loading with DHA (Fe-MnO_2_/DHA), and found Fe-MnO_2_/DHA could trigger multi-modal programmed cell deaths in liver cancer. Moreover, in contrast with free DHA, the functional outcome of DHA-loaded nanoscale carriers is dramatically enhanced for cancer treatment [23]. Similarly, we constructed a self-assembled MOF NPs based on zeolitic imidazolate framework-8 to load with DHA (ZIF-DHA). The in vitro and in vivo assays indicated that, compared with the free DHA, ZIF-DHA administration possess preferable therapeutic activity against ovarian cancer (Fig. 8).

ROMO1 is localized to the inner mitochondrial membrane, and functions as a redox-sensitive protein that regulates the mitochondrial homeostasis and cellular ROS generation [24]. Subsequent studies have proved the underlying roles of aberrant ROMO1 in the cancer development and treatment. Through evaluating the profiles of ROMO1 in cancer samples, several groups independently demonstrated that ROMO1 could be served as a promising diagnostic and prognostic biomarker [25–27]. Liu et al. found that overexpression of lncRNA TUG1 could promote the promoter function and transcriptional expression of ROMO1, leading to the improved cell proliferation and metastasis in hepatocellular carcinoma cells Huh7 [28]. In glioma, knockdown of ROMO1 inhibited the cell cycle progression and cell growth by decreasing cellular ROS production [29]. In addition, through controlling the generation of ROS, ROMO1 inhibition significantly triggered the apoptotic cell death in colorectal cancer cells [30]. Similarly, in our mechanism analysis, 4D-FastDIA-based mass spectrometry technology revealed that downregulated ROMO1 might be the potential target for ZIF-DHA treatment. ZIF-DHA seem to effectively decrease the ROMO1 expression, generating the apoptotic cell death in ovarian cancer cells.

During tumorigenesis, different stresses such as metabolic stress and oxidative stress could induce the aberrant hyper-proliferation signals. Stress-induced ROS accumulation displays the pivotal roles in tumor progression and therapeutic response through dictating a multitude of signaling pathways [31, 32]. Nowadays, several ROS generation strategies have been developed to achieve the improved efficacy of anti-cancer therapy. Under excessive ROS conditions, the ROS responsive nanoagents could be effectively absorbed by cancer cells, leading to the cuproptosis and anti-cancer immune response [33]. Moreover, MOF@Au nanoreactor has been proved to be an oxidative stress amplifier. After entering into cancer cells, MOF@Au nanoreactor could significantly stimulate the ROS formation and amplify the oxidative damage to cancer cells [34]. Surprisingly, the ovarian cancer cells frequently exhibit the fragmented and fissile mitochondria, contributing to cellular ROS generation and cell growth [35]. Activation of mitofusin 2 (Mfn2) by genetic or pharmacological tools restrained the mitochondrial fission and reduced ROS concentration, subsequently suppressing ovarian cancer progression [36]. Accordingly, we utilized the zeolitic imidazolate framework-8-based MOF to encapsulate DHA to form the ZIF-DHA NPs. Subsequent functional analyses showed that ZIF-DHA could be stably absorbed into the ovarian cancer cells, leading to the decreased ROS generation and increased tumor-killing effect.

In conclusion, we successfully constructed and characterized the potential anti-cancer mechanisms of ZIF-DHA NPs, for enhancing the therapeutic outcome of DHA in human ovarian cancer. These findings demonstrated that ZIF-DHA could be the useful anti-cancer candidates for the control of ovarian cancer. Moreover, identification of nanoscale carriers, like MOF, that load with DHA might provide the most promising strategies for the eradication of ovarian cancer.

## Electronic supplementary material

Below is the link to the electronic supplementary material.

Figure S1. The hydordynamic size within 48 h was assayed to inciated the stability of prepared ZIF-DHA

Figure S2. The TEM revealed the collapse of nanoagents in acidic microenvironment

Figure S3. The underlying cytotoxic effects of ZIF-DHA against several human cancer cells indicated by CCK-8

Figure S4. The quantitative analysis for co-localization efficient of ZIF-DHA and mitochondria

Figure S5. The concentrations of zinc ions in tumor grafts and vital organs were detected using ICP

Table S1. The loading efficiency of DHA on ZIF NPs

Figure S6. The levels of zinc ions in the blood circulation within 24 h were measured by ICP

Figure S7. The morphology of vital organs was observed by hematoxylin-eosin staining

Table S2. The identified proteins by mass spectrometry

Table S3. The differentially expressed proteins in ZIF-DHA-treated A2780 cells

Table S4. The differentially expressed proteins in ZIF-DHA-treated SKOV3 cells

Table S5. The co-differentially expressed proteins in ZIF-DHA-treated cells

## Data Availability

The data used to support this review are included within the article.

## Abbreviations

NPs: nanoparticles
DHA: dihydroartemisinin
MOF: metal-organic frameworks
ROS: reactive oxygen species
TEM: transmission electron microscopy
PVDF: polyvinylidene fluoride
GSEA: gene set enrichment analysis
H_2_O_2_: hydrogen peroxide
ROMO1: reactive oxygen species modulator 1
ERK: extracellular signal-regulated kinase
AKT: protein kinase B
Mfn2: mitofusin 2
