# Supplementary figures and images for: Metal-organic framework-encapsulated dihydroartemisinin nanoparticles induces apoptotic cell death in ovarian cancer by blocking ROMO1-mediated ROS production
Source: J Nanobiotechnology. 2023 Jun 29;21:204. doi: 10.1186/s12951-023-01959-3 (PMC10308639; doi:10.1186/s12951-023-01959-3)

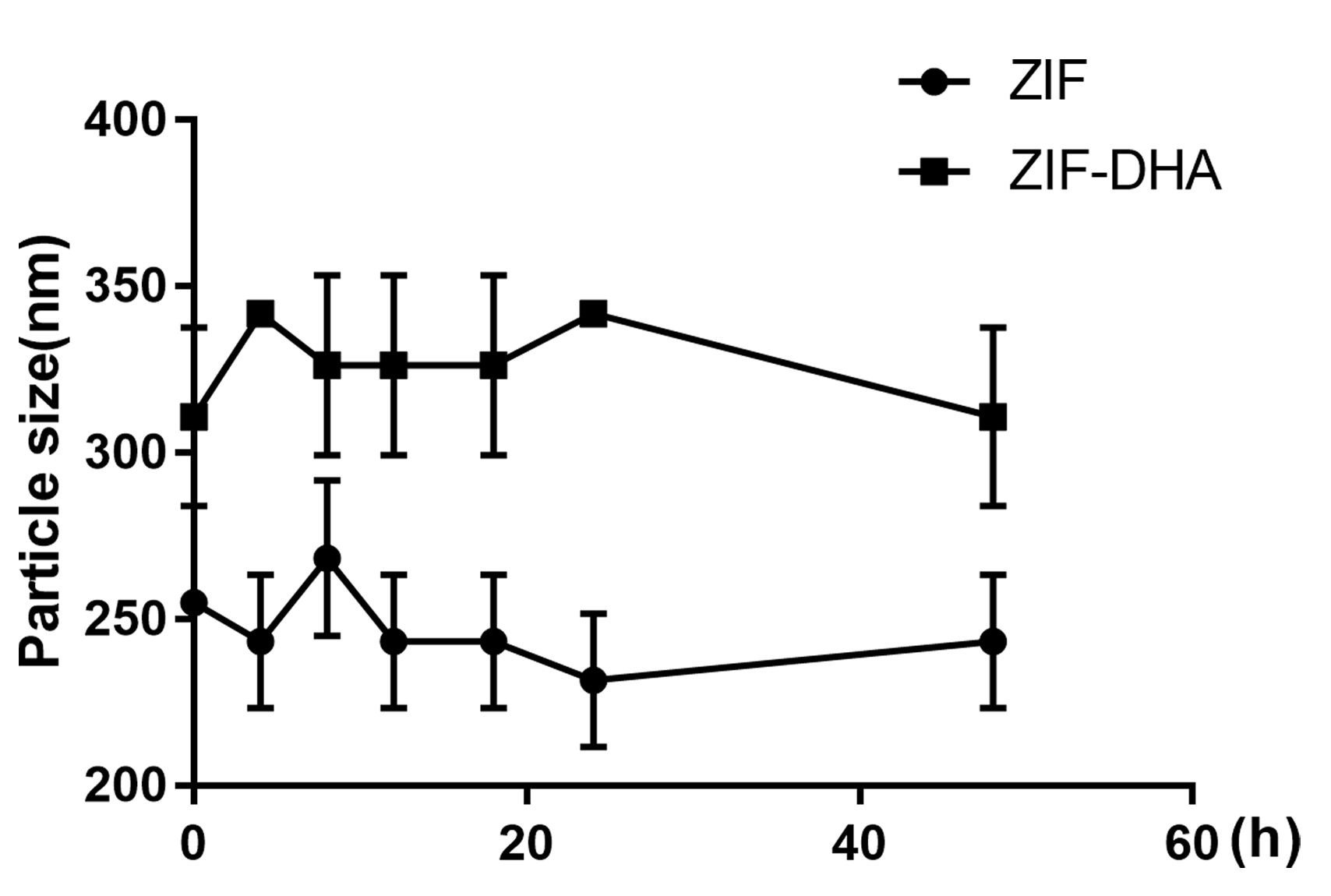

Supplement: Supplementary file 1 — Figure S1. The hydordynamic size within 48 h was assayed to inciated the stability of prepared ZIF-DHA [file 12951_2023_1959_MOESM1_ESM.tif]

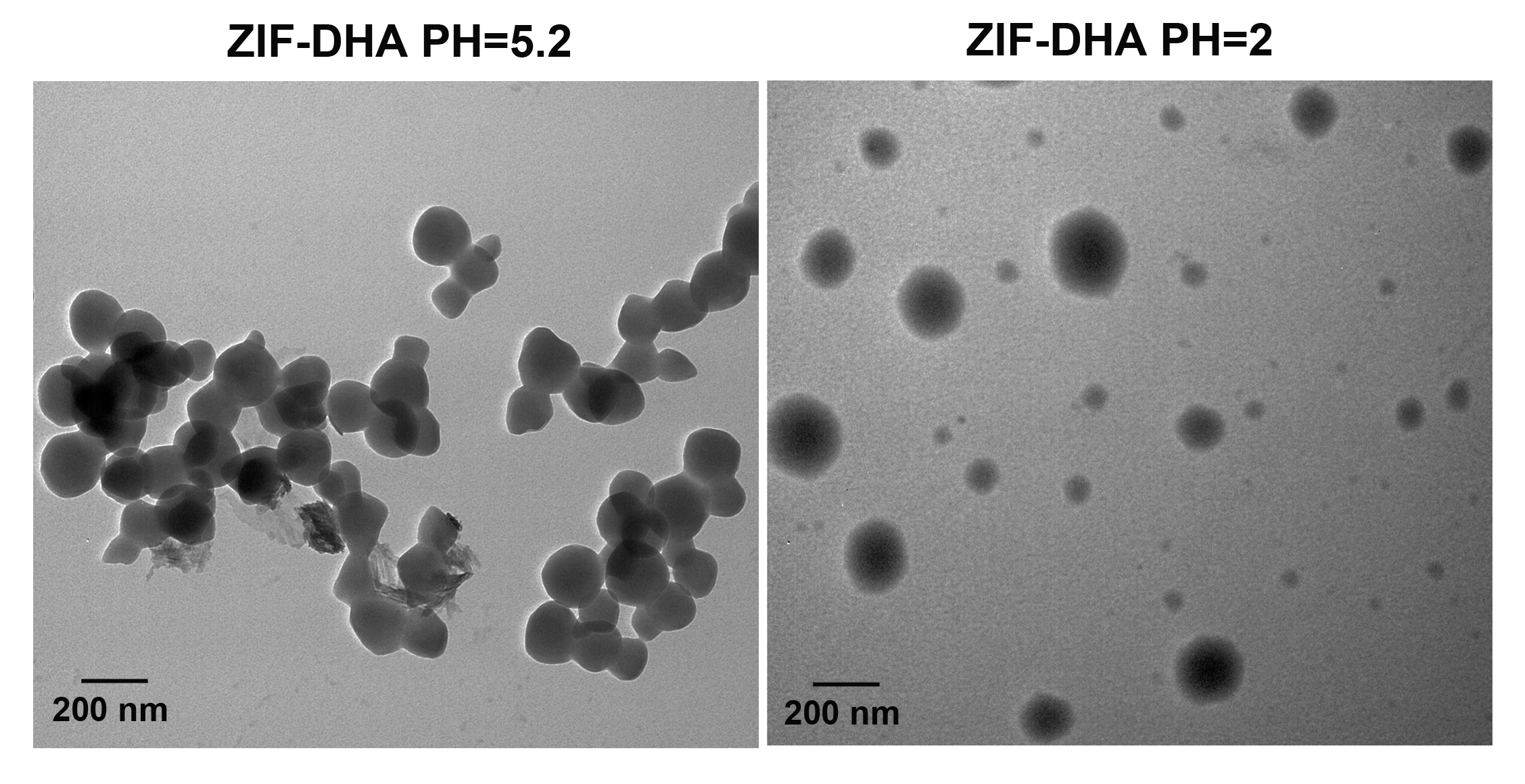

Supplement: Supplementary file 2 — Figure S2. The TEM revealed the collapse of nanoagents in acidic microenvironment [file 12951_2023_1959_MOESM2_ESM.tif]

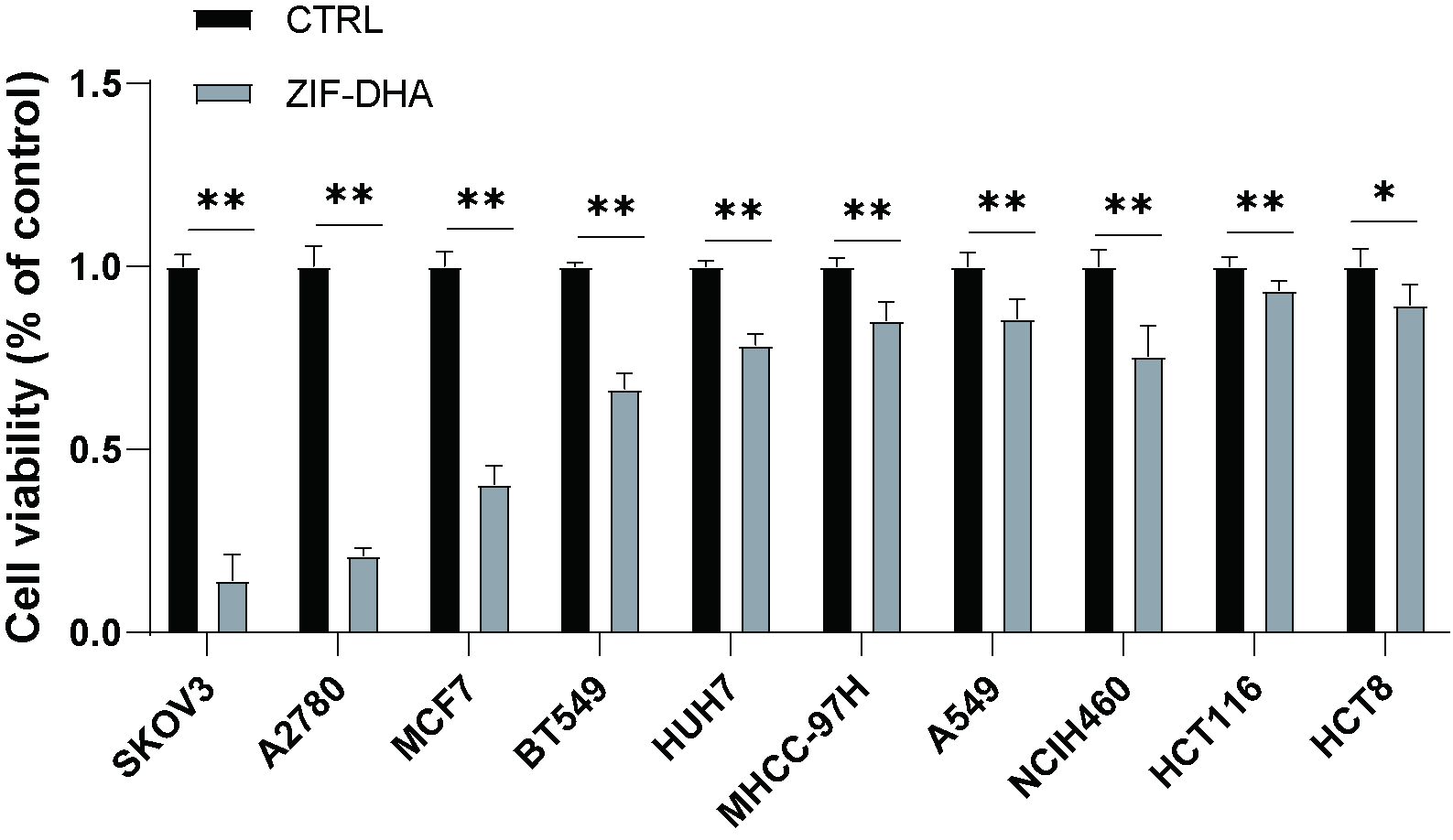

Supplement: Supplementary file 3 — Figure S3. The underlying cytotoxic effects of ZIF-DHA against several human cancer cells indicated by CCK-8 [file 12951_2023_1959_MOESM3_ESM.tif]

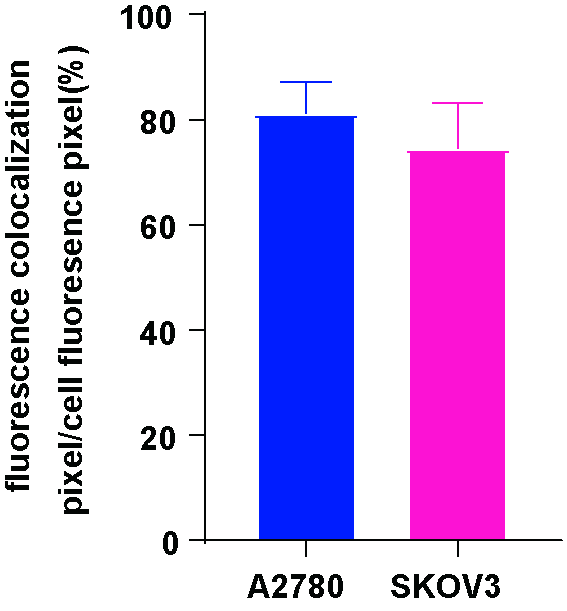

Supplement: Supplementary file 4 — Figure S4. The quantitative analysis for co-localization efficient of ZIF-DHA and mitochondria [file 12951_2023_1959_MOESM4_ESM.tif]

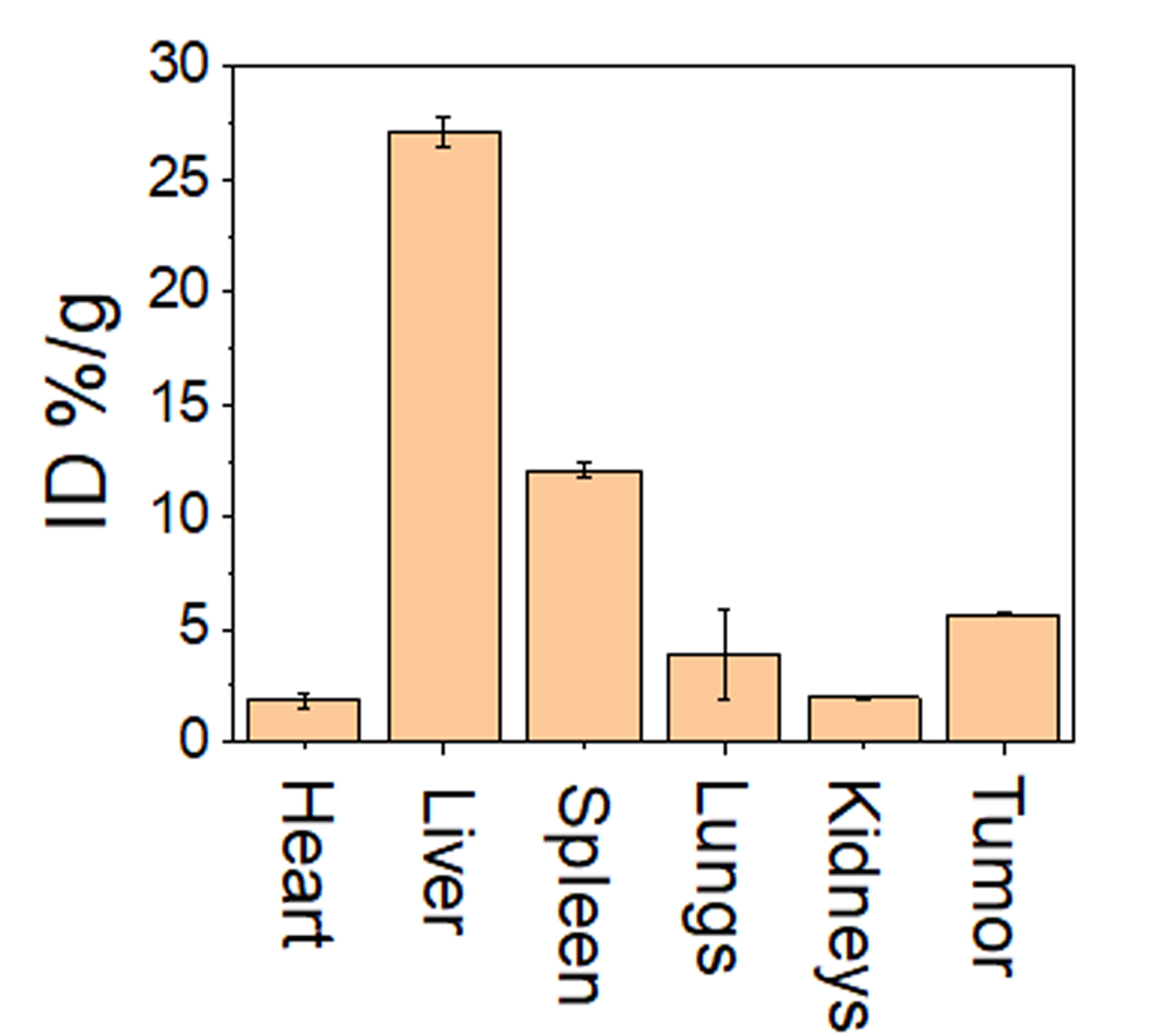

Supplement: Supplementary file 5 — Figure S5. The concentrations of zinc ions in tumor grafts and vital organs were detected using ICP [file 12951_2023_1959_MOESM5_ESM.tif]

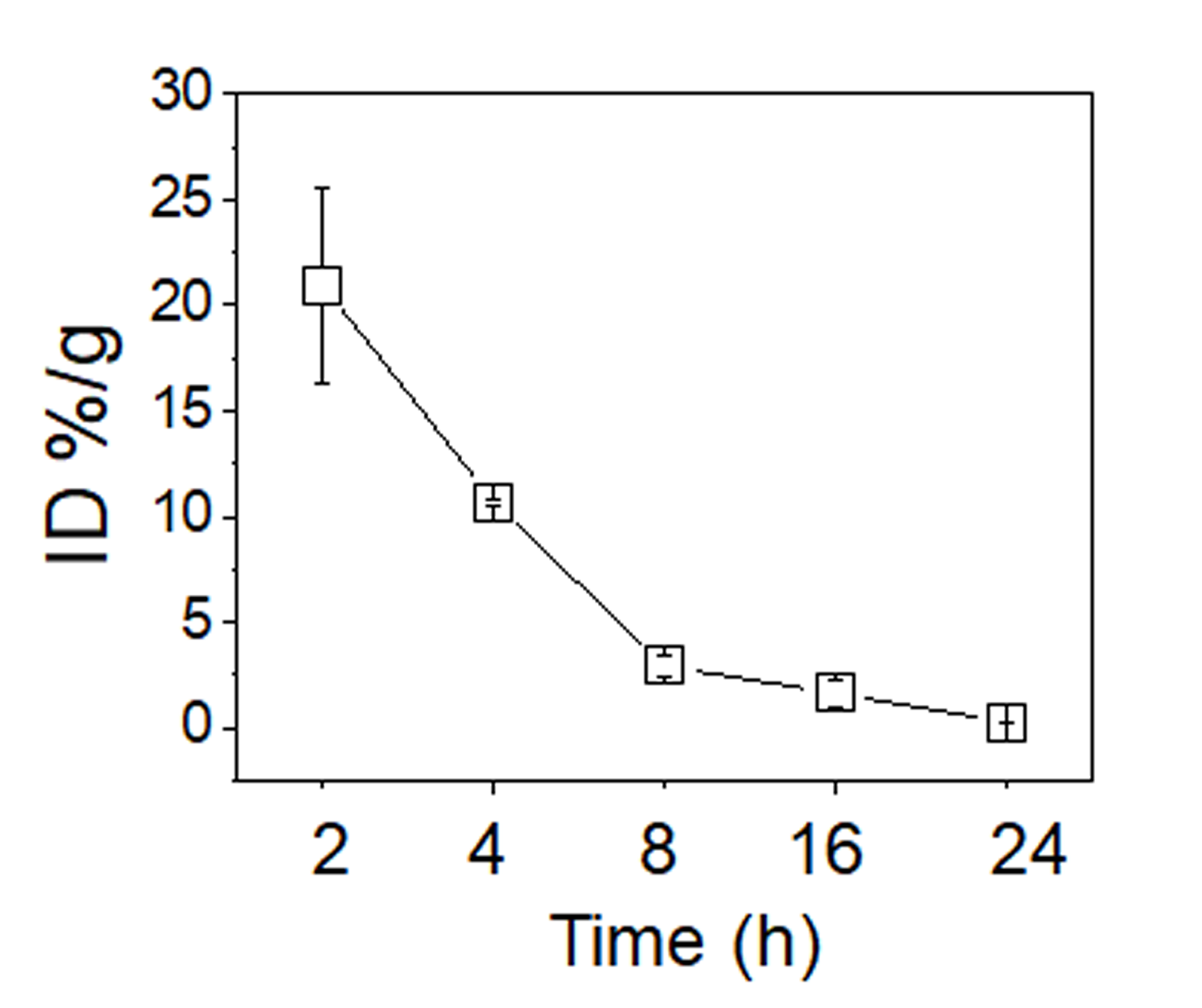

Supplement: Supplementary file 7 — Figure S6. The levels of zinc ions in the blood circulation within 24 h were measured by ICP [file 12951_2023_1959_MOESM7_ESM.tif]

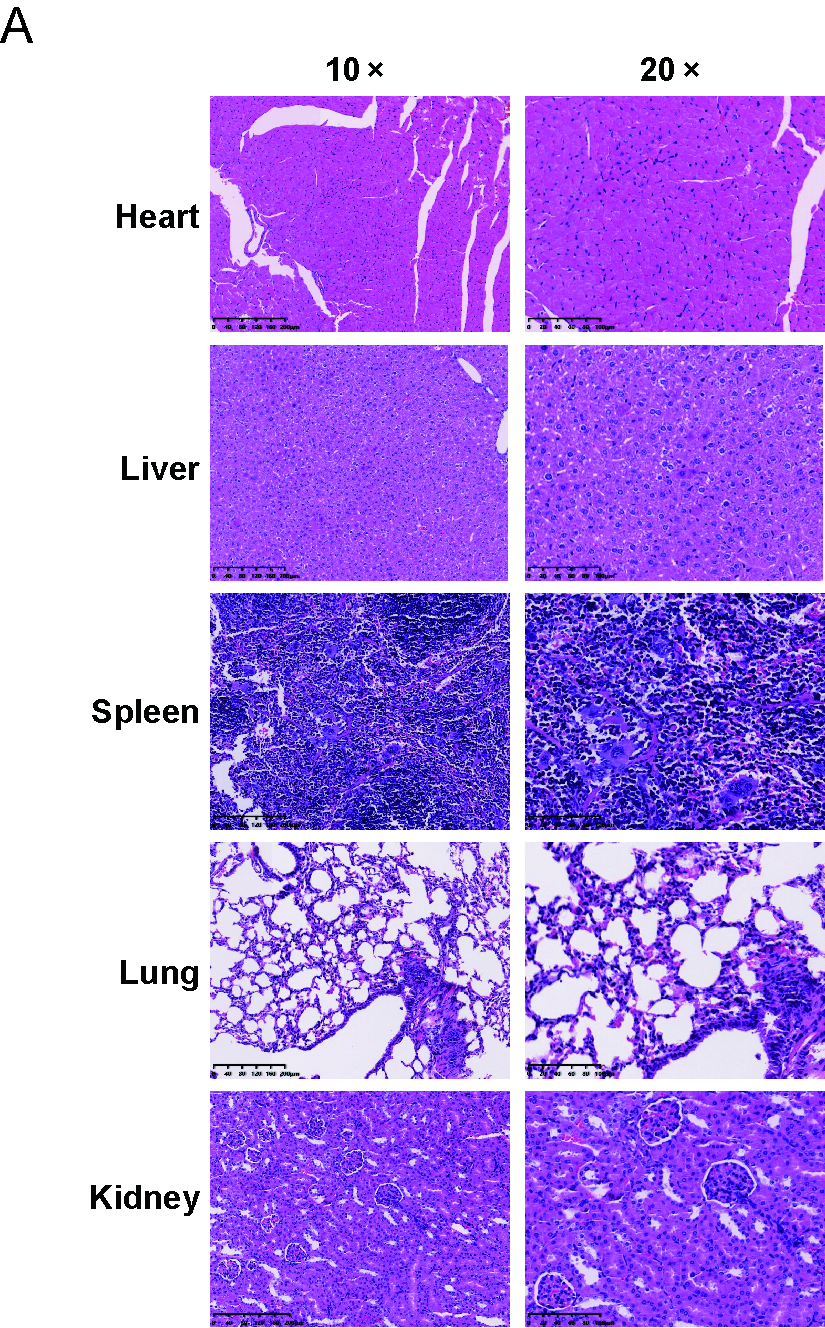

Supplement: Supplementary file 8 — Figure S7. The morphology of vital organs was observed by hematoxylin-eosin staining [file 12951_2023_1959_MOESM8_ESM.tif]
